# The Chinese government's response to drug use and HIV/AIDS: A review of policies and programs

**DOI:** 10.1186/1477-7517-7-4

**Published:** 2010-03-05

**Authors:** Jianhua Li, Toan H Ha, Cunmin Zhang, Hongjie Liu

**Affiliations:** 1 Yunnan Institute for Drug Abuse, Yunnan, China; 2 Department of Epidemiology and Community Health, School of Medicine, Virginia Commonwealth University, Richmond, Virginia, USA

## Abstract

Illicit drug use has become popular in China. Acknowledging the challenge of illicit drug use, China has adopted several new policies on the management of illicit drug use in recent years. This study reviews the current policies on drug use and assesses the harm reduction interventions among drug users in China. The review documents that the new policies on drug use provide a variety of choices of detoxification treatment for drug users. The methadone maintenance treatment and needle exchange programs have been adopted as harm reduction models in China. Most of the reviewed harm reduction programs have been successfully implemented and yielded positive effects in reducing drug related risk behaviors among drug users. Although there remain barriers to the effective implementation of policies on drug use and harm reduction programs, Chinese government has shown their commitment to support the expansion of harm reduction interventions for drug users throughout the country.

## Introduction

### History of drug use in China

China has a long history of illicit drug use. Opium was first trafficked into China by ancient Arabians in early 700 BC [1]. During the 16th century, British colonists imported Indian opium into China as a means of exchange for goods such as silk, tea and cash. Since then, opium has been grown and used throughout China [2]. The number of drug addicts exceeded 20 million in 1949 when the new China was founded. Following the launching of an extensive anti-drug campaign in the early 1950s, the Chinese government news agency announced in 1953 that drug abuse was completely eliminated from the mainland, which led China to be considered a drug-free country for the next thirty years [2,3].

Drug use re-emerged in China as it adopted economic reforms and an open door policy to the outside in 1980s. The drug trafficking activities were carried out mainly through the route from the Golden Triangle region (Myanmar, Yunnan and Guizhou provinces) to Guangzhou and Hong Kong [4]. The large amount of heroin and opium in the Chinese market has been brought from Myanmar into Yunnan Province or from Viet Nam into Guangxi Province [5]. According to the World Drug Report in 2008, China had the third largest heroin seizures in the world and the largest cocaine seizures in Asia in 2006 [6].

According to the China Ministry of Public Security, the number of registered drug users increased from 70,000 in 1990 to 1.16 million in 2005 [7,8], however, the estimated number is believed to be higher [9]. The majority of drug users use heroin. Most of them are young, have little education and do not have stable employment [10,11]. New drugs including amphetamine-type-stimulants (ATS), particularly methamphetamine, and other narcotics have become popular among youth since late 1990s and have since become available in medium- and large- sized cities in China [9,12].

### Brief overview of HIV/AIDS infection in injecting drug users

The HIV epidemic in China began in 1989 among injection drug users (IDUs) [13]. Injection drug use (IDU) was the major HIV transmission route in the country, with cases of HIV/AIDS diagnosed among drug users in all 31 provinces, municipalities and autonomous regions by 2002 [14]. According to a recent estimate, 44.7% of 50,000 new HIV cases in 2007 were infected through heterosexual contact, and 42% through IDU [15]. The latest estimate of HIV prevalence among IDUs is 12.55% [16]. However, HIV infection varies from region to region. The HIV infection rates among IDUs in Xinjiang, Guangxi and Sichuan provinces are 41.31%, 16.95% and 15.12% respectively; whereas, HIV infection rates among IDUs in Xingjiang and Yunnan province reached to 52.51% [16]. As the number of drug users continues to increase and the HIV epidemic continues to spread in this population, IDU remains the major cause of HIV infection [17].

Despite that the Chinese government has enacted several policies and programs in response to drug users and HIV prevention, the review of these policies and related harm reduction programs has not been thoroughly conducted. The purpose of this paper was to review the current policies against drug use and harm reduction programs in China. The results of the review will not only provide information for the Chinese government to better respond to the HIV epidemic among drug users, but also provide recommendations for future research

## Methods

The following criteria were used in the review: (1) studies written in English and published in international peer-reviewed journals between 1990 and 2008, (2) harm reduction intervention studies among drug users in mainland China and (3) related government policies on illicit drug abuse and HIV intervention programs.

Relevant intervention studies were obtained using the following databases: Pubmed, Medline, EBSCO and Google Scholar. The combinations of key words used to identify relevant articles included: China, HIV, AIDS, drug use, narcotic control, incarceration, voluntary counseling and testing and harm reduction intervention.

## Results

### Current policies regarding drug use and HIV prevention among drug users

In 1990, the Standing Office of National People's Congress enacted "Regulations on Prohibition against Narcotics" which specified three levels of management. In the first, drug users are fined and/or encouraged to receive treatments in voluntary detoxification centers managed by the Ministry of Public Health [18]. They usually stay in the centers for 7-30 days [7,19]. Those who have gone through the first level and relapse are sent to compulsory detoxification centers administered by the Public Security Bureau (second level) [5,7,19-22]. They spend at least 6 months in the compulsory detoxification centers where they participate in a combination of detoxification treatment, physical exercise, and manual labor [7,19-22]. At the third level, drug users who relapse after receiving compulsory detoxification are mandated to 2 to 3 years of re-education through activities in labor camps administered by the Justice Bureau [19,21,23]. As drug users are not allowed to leave the compulsory detoxification centers and labor camps, they are considered incarceration sites.

As stated in the "Regulations on Prohibition against Narcotics", the main purpose of the compulsory and voluntary detoxification is to reduce demands for drug use, drug related crimes, and injection drug use, which are designed to eventually reduce HIV transmission rates [21]. The Ministry of Health has issued guidelines on the selection of detoxification medicines, but the actual treatment regime and quality of the treatment vary across centers. Psychosocial counseling and behavioral intervention services for drug users are largely unavailable in the detoxification centers, mainly because they do not have enough professional staff and funding to provide these services, especially at the city or county level. Police and medical staff lack the knowledge and skills for delivering harm-reduction and behavioral-change counseling. Police often apply military management approaches to treat drug users while they are in incarceration sites. The majority of treated drug users relapse and do not change their HIV risk behaviors after they leave incarceration sites [21].

In 2006, the Chinese Ministry of Public Security issued a policy program called " The treatment of arrested drug users in Compulsory Detoxification Centers". According to this policy, newly-found drug users were sent directly to compulsory detoxification centers, while relapsed drug users were to reeducation labor camps [24]. The Ministry of Public Security and the Department of Public Security at the provincial and city levels initiated a nationwide crackdown on drug use and an intensified compulsory detoxification program for drug users. In June 2006 alone, 269,000 drug users were incarcerated and 71,000 of them were sent to reeducation labor camps [24]. At the end of 2006, there were about 1000 incarceration sites in China. Over 600,000 drug users have been admitted to incarceration sites, 95% of whom were heroin users and 3% were opium users (Jian, unpublished data).

The newly-promulgated "Narcotic Control Law" that took effect on June 1, 2008 [25] had significant reforms on the management of drug abuse in China. The new law defines a clear leadership system, and a working mechanism and supporting system for narcotics control. It specifies that narcotics control is the responsibility of the whole society, including governmental agencies at all levels and all Chinese people. The law requires the State Council to establish the National Narcotics Control Commission, and the local governments at or above the county level to set up a narcotics control committee which organizes, coordinates and guides narcotics control activities in their jurisdictional areas. The establishment of the commission and the committees demonstrates that the Chinese government has intensified its efforts to curb drug abuse. In contrast to previous policies which addressed the detoxification treatment system, the new law prioritizes prevention and intervention activities in association with comprehensive drug control models and simultaneous efforts to ban the cultivation, production, trafficking, and abuse of drugs. The law introduces significant reforms on drug treatment and rehabilitation in accordance with the human-oriented principle. According to the new law, drug users are not required to stay in detoxification centers, rather, they have the right to select treatment or/and other services from authorized institutions that provide these services. In addition, the law also orders that drug users' rights be protected. They have the same rights to education, jobs, and social support [25].

In confronting the HIV/AIDS epidemic, China has adopted the methadone maintenance treatment (MMT) and needle exchange program (NEP) as harm reduction models. The *Five Year Action Plan to Control HIV/AIDS 2006-2010 *issued by the Ministry of Health included both MMP and NEP [23]. Methadone treatment has been used as a detoxification method in China since 1993 [23], but its use was limited to inpatients in well-equipped institutions [26]. Recognizing the efficacy of MMT for drug dependence, China supported the use of MMT to palliate HIV transmission in 2004 [27-29]. The first eight government-supported pilot MMT clinics were established in five provinces in early 2004 [23,30]. The program has since grown to 320 clinics, serving a total of 27,000 heroin users in 22 provinces [28]. By November 30, 2008, 558 MMT clinics had been put into operation in 23 provinces, autonomous regions, and municipalities, serving more than 170,000 clients [31].

The NEPs were first initiated by non-governmental organizations (NGOs) and other international donors rather than by the Chinese government because of the concern that these programs condoned illicit drug use [32]. When first introduced in China, NEPs were called social marketing of needle exchange, which aimed to promote commercial availability and accessibility of needles in combination with health education about safe injecting practice, a concept approved by the Ministries of Health and Pubic Security because it did not explicitly mention needle exchange or free needle distribution [28,33]. Acknowledging the growing research evidence of successful NEPs in other countries, the Ministry of Health officially funded the first pilot NEP in Guangxi and Yunnan province in 1999 [23,27,28]. The program has expanded to other parts of China, with a total of 790 needle exchange centers, 392 of which were funded by the government in 2006 [34]. The Chinese central government incorporated NEPs into the second five-year action plan for HIV intervention (2006-2010) and mandated that enough NEP sites be established to serve at least 50% of the IDU population by 2010 [34].

### Results of harm reduction interventions and HIV prevention among drug users in China

The results of the empirical studies [5,35-41] in China revealed that most of the interventions on harm reduction (e.g. MMT and NEP) and HIV prevention (e.g. VCT) were successful and had positive effects in reducing drug related risk behaviors among drug users in both institution-based and community-based programs. For example, two MMT intervention studies showed that the use of MMT decreased the frequency of IDU and criminal behaviors [37]. Three needle related studies revealed a significant reduction of needle-sharing among drug users [40,41] and a decline of new drug injectors in the follow-survey [39]. Results of VCT studies documented that participants increased their HIV/AIDS knowledge [35], increased condom use with regular and casual sex partners, and decreased needle-sharing [36]. Two HIV knowledge-oriented studies have shown that participants, after receiving the intervention, increased their HIV-related knowledge, their understanding of HIV prevention methods, and indicated positive attitudes toward those with HIV/AIDS [38,42]. These positive results are consistent with the large body of empirical evidence on effectiveness of harm reduction programs in other countries [43-49].

## Discussion

China has made substantial progress in the development and implementation of policies on the management of drug use and on effective intervention strategies for HIV/AIDS in the past few years [27]. The newly-enacted Narcotic Control Law is a milestone in harm reduction and HIV prevention initiative. With the hierarchical authority system in China, the new law would be quickly enacted at different administration levels, ushering in a new era of drug management and HIV intervention in both community level and incarceration sites in the world's most populous nation.

Given the positive effects of MMT among drug users, China is offering long-term use of MMT to palliate HIV transmission in 2004 [27-29]. However, the conflicting approach between "zero tolerance" policies toward drug use and harm reduction programs [23], increasing the demand for care, support and treatment for IDUs to reach those in need [31] and a lack of cooperation among departments at local levels (e.g., at the county level or below) may make it difficult for the effective implementation of harm reduction programs [50]. A synchronized drug use control approach, open communication and strong cooperation among involved governmental departments are needed to ensure the effective implementation of the harm reduction initiatives in China. As a substantial number of drug users are incarcerated and a proportion of them are HIV positive, behavioral interventions should be implemented in incarceration centers to prevent further spread of HIV.

The new Narcotic Control Law demonstrates China's changed approach toward drug users. It reflects a humane and people-first principle and provides a variety of choices of detoxification treatment for drug users. The implementation of this law requires not only strong central government support, multi-sectoral participation and collaboration, but also a strong commitment from the local authorities. The central government needs to provide clear guidelines for implementation of the new law and make sure that these guidelines are strictly followed by all local governments. While the new law offers a comprehensive approach toward drug use, no empirical evidence on the effectiveness of its implementation has been available. Evaluation of its effectiveness needs to be conducted.

Despite attempts by the Chinese central government to include NEPs into the second five-year action plan (2006-2010) (State Council of P.R. China, unpublished data), NEPs have not been fully supported by all governmental agencies. The Ministry of Public Security does not support the implementation of NEPs [27]. This perspective has created a challenge for public health workers who implement NEPs at the local levels [5]. The central government should coordinate different ministries and departments to work on this conflict. The sustainability and effectiveness of NEPs program can only be obtained if there is full support from all participating government agencies in China.

At the program level, findings of the review of NEPs revealed that there remain barriers to access to NEPs including the long distance to NEP sites, difficult access to the service, and fear of being arrested when receiving new needles [41]. Therefore, not only the coverage of NEPs should be expanded, but also drug users must be assured that it is safe to receive needles and that they should not fear of the arrest by police. The Public Security Bureau at local levels should be made aware of evidence demonstrating the positive effects of NEPs in reducing HIV transmission among IDUs and that NEPs do not increase drug use [44,45,51].

Although the government has mandated that 50% coverage of injection drug users by NEPs, the coverage target is insufficient. Several studies show that low syringe coverage can severely affect the effectiveness of NEPs [52,53]. For example, lack of syringe coverage might have contributed to the limited effectiveness of NEPs in Montreal [53]. Therefore, the government should increase higher syringe coverage targets for injection drug users and allocate funding for NEPs in the annual budget plan.

Available literatures show that peer education plays an effective role in the harm reduction programs [54,55]. Although peer education has been conducted successfully in a number of areas in China, most of the interventions have been restricted to small locally based programs [56,57]. Large-scale peer education programs have not been conducted [58]. Therefore, the government should not only support for the development of a national education program, but create favorable environments for the program implementation.

The existing intervention programs are largely run by government agencies. NGOs in China should be encouraged to actively participate in drug and HIV intervention programs. Only one VCT intervention study among drug users has been successfully implemented by an NGO and published in China so far [36]. The success of this NGO-led intervention program suggests that harm reduction programs delivered by NGOs should be expanded in China. NGOs have advantages in drug and HIV intervention over government agencies as they are able to reach out high risk groups (e.g. IDUs or sex workers) without making them fear of arrest or stigmatization [18]. Experiences from other countries demonstrate that NGOs play an important role in controlling and preventing HIV/AIDS [59,60].

Although results of the available studies are encouraging, few studies reported the application of a theoretical framework for the intervention programs. Studies from other countries have demonstrated that theory-based intervention programs have proven effective in reducing HIV related risk behaviors among drug users [61,62]. A recent review of HIV behavioral interventions for a US high-risk population in 2000-2004 found that all best-evidence interventions relied on at least one behavioral change theory or model [63]. Further empirical evidences have shown that adding behavioral intervention (e.g. VCT, health education and health promotion) components into NEP and MMT programs results in reducing injecting-related risk practices (e.g. sharing needles), and decreasing high-risk sexual behaviors (e.g. unprotected sex) [64-67]. Theory-based harm reduction interventions are recommended to promote behavior change among drug users in China. Psychological and behavioral counseling services should be integrated with existing harm reduction programs.

## Conclusions

The Chinese government has made significant progress in evolving the policy on drug abuse. Policies on harm reduction and HIV intervention have been improved and enhanced based on the findings from research and practices. This review demonstrates that harm reduction programs have been successfully implemented in most cases and yielded positive effects in reducing drug related risk behaviors among drug users in China. This evidence serves as an important resource for advocating the further development of harm reduction programs among this population. Despite the fact that there remain challenges, China is increasingly adopting scientific evidence-based approaches and has encouraged the pilot testing of methods of risk reduction [27]. This increasing use of scientifically validated evidence together with supportive policy on harm reduction programs, demonstrates not only the Chinese government's continuing commitment and determination in the fight against HIV, but also the importance of future intervention research in China.

## Lists of abbreviations

ART: antiretroviral therapy; ATS: amphetamine-type-stimulants; HIV: human immunodeficiency virus; IDU: injection drug use; IDUs: injecting drug users; MMT: methadone maintenance treatment; NNCC: National Narcotics Control Commission; NEP: needle exchange program; NGO: non-governmental organization; VCT: voluntary counseling and testing.

## Competing interests

The authors declare that they have no competing interests.

## Authors' contributions

JL conceived the study, CZ and THH carried out the literature search. JL, HL and THH wrote the manuscript. All authors read and approved the final manuscript.

## References

[B1] Shu ZL History of Drug Abuse in China 1995 Shanghai: Shanghai People Publishing House

[B2] McCoy CB McCoy HV Lai SH Wang X Meng J Reawakening the dragon: Changing patterns of opiate use in Asia, with particular emphasis on China's Yunnan Province Subst Use Misuse 2001 36 49 69 10.1081/JA-100000228 11305354

[B3] Lowinger P The Solution to Narcotic Addiction in the People's Republic of China Am J Drug Alcohol Abuse 1997 4 165 178 10.3109/00952997709002758 347925

[B4] Naik TN Sarkar S Singh HL Bhunia SC Singh YI Singh PK Pal SC Intravenous drug users-a new high-risk group for HIV infection in India AIDS 1991 5 117 118 2059356 10.1097/00002030-199101000-00026

[B5] Qian HZ Hao C Ruan Y Cassell HM Chen K Qin G Yin L Achumacher JE Liang S Shao Y Impact of methadone on drug use and risky sex in China J Subst Abuse Treat 2008 34 4 391 397 10.1016/j.jsat.2007.07.002 17869049

[B6] Office on Drugs and Crime, United Nations 2008 World Drug Report 2008 1 310 http://www.unodc.org/documents/wdr/WDR_2008/WDR_2008_eng_web.pdf

[B7] Chu TX Levy JA Injection drug use and HIV/AIDS transmission in China Cell Research 2005 15 865 869 10.1038/sj.cr.7290360 16354561

[B8] IHRD Harm Reduction Developments 2008 New York: International Harm Reduction Development Program of the Open Society Institute

[B9] Kulsudjarit K Drug problem in southeast and southwest Asia Ann N Y Acad Sci 2004 1025 446 457 10.1196/annals.1316.055 15542748

[B10] Liu Z Lian Z Zhao C Drug use and HIV/AIDS in China Drug Alcohol Rev 2006 25 2 173 176 10.1080/09595230500538835 16627308

[B11] Fu XB Lin P Li J Epidemiological survey on poly-drug abuse in intravenous drug users in Guangdong Province South China Journal of Preventive Medicine 2004 30 8 11

[B12] Lu L Fang Y Wang X Drug abuse in China: Past, Present and Future Cell Mol Neurobio 2008 28 479 490 10.1007/s10571-007-9225-2 PMC11515005 17990098

[B13] Wang L Overview of the HIV/AIDS epidemic, scientific research and government responses in China AIDS 2007 21 S8 S3 S7 10.1097/01.aids.0000304690.24390.c2 18172388

[B14] UNAIDS 2004 report on the global AIDS epidemic 2004 Geneva: UNAIDS

[B15] Wang L Wang N Wang L Li D Jia M Gao X Qu S Qin Q Wang Y Smith K The 2007 Estimates for People at Risk for and Living With HIV in China: Progress and Challenges J Acquir Immune Defic Syndr 2009 50 4 414 418 10.1097/QAI.0b013e3181958530 19214116

[B16] Bao PY Liu MZ Systematic review of HIV and HCV infection among users in China Int J STD AIDS 2009 20 339 405 10.1258/ijsa.2008.008362 19451325

[B17] Ministry of Health, UNAIDS & WHO 2005 update on the HIV/AIDS epidemic and response in China http://data.unaids.org/publications/External-Documents/rp_2005Chinaestimation_25jan06_en.pdf

[B18] Qian H Schumacher EJ Chen TH Ruan Y Injection drug use and HIV/AIDS in China: Review of current situation, prevention and policy implications Harm Reduct J 2006 16451717 10.1186/1477-7517-3-4 PMC1402269

[B19] Zhao C Liu Z Zhao D Liu Y Liang J Tang Y Liu Z Zheng J Drug abuse in China Ann YY Acad Sci 2004 1025 439 445 10.1196/annals.1316.054 15542747

[B20] Zhao B Liu X Studies in drug related crime in Beijing 1993 Beijing: Chinese People's University Press

[B21] Jianhua LT Challenges for China's present drug detoxification work China Journal of Drug Dependence 2004 13 224 226

[B22] Wang W Illegal drug abuse and the community camp strategy in China J Drug Educ 1999 29 97 114 10.2190/J28R-FH8R-68A9-L288 10429353

[B23] Lu L Wang X Drug addiction in China Ann N Y Acad Sci 2008 1141 307 317 10.1196/annals.1441.025 18991965

[B24] National Narcotics Control Commission (NNCC) Annual Report on Drug Control in China 2007 Beijing: NNCC

[B25] Significance and Content of the Narcotics Control Law. Office of China National Narcotics Control Commission http://www.China.org.cn/e-news/news080625-3.htm

[B26] Tang Y Zhao D Zhao C Cubells FJ Opiate addiction in China: current situation and treatments Addiction 2006 101 657 665 10.1111/j.1360-0443.2006.01367.x 16669899

[B27] Wu Z Sullivan GS Wang Y Rotheram-Borus JM Detels R Evolution of China's response to HIV/AIDS Lancet 2007 369 679 690 10.1016/S0140-6736(07)60315-8 17321313 PMC7137740

[B28] Sullivan GR Wu Z Rapid Scale up of harm reduction in China Int J Drug Policy 2007 18 118 128 10.1016/j.drugpo.2006.11.014 17689354

[B29] Bates G Morrison JS Thompson D China's time bomb: sustaining the momentum of China's HIV/AIDS response. A report of the CSIS HIV/AIDS delegation to China, April 13-18 CSIS - Center for Strategic and International Studies. Washington, D.C 2004 http://www.hivpolicy.org/Library/HPP000649.pdf

[B30] Hammett TM Wu Z Duc TT Stephen D Sullivan S Liu W Chen Y Ngu D Des Jarlais DC " Social evils" and harm reduction: the evolving policy environment for HIV prevention among injecting drug users in China and Vietnam Addiction 2007 103 137 145 10.1111/j.1360-0443.2007.02053.x 18028519

[B31] Lin C Wu Z Rou K Yin W Wang C Shoptaw S Detels R Structural-level factors affecting implementation of the methadone maintenance therapy program in China J Subst Abuse Treat 2010 38 119 127 10.1016/j.jsat.2009.09.002 20015606 PMC2814942

[B32] Ma Y Wu Z Campaign against drugs and control of IDU transmitted HIV/AIDS in China Zhongguo Xing Bing Ai Bing Fang Zhi 2000 6 185

[B33] Yap L Wu Z Liu W Ming XQ Liang S A rapid assessment and its implications for a needle social marketing intervention among injecting drug users in China Int J Drug Policy 2002 13 57 68 10.1016/S0955-3959(01)00118-9

[B34] State Council of People's Republic of China China's action plan for reducing and preventing the spread of HIV/AIDS (2006-2010) 2006 Beijing: State Council of People's Republic of China

[B35] Grusky O Liu H Li X Swangson A Duan N Is Voluntary Counseling and Testing of Drug Users in China Feasible Int J STD AIDS 2006 17 354 355 10.1258/095646206776790196 16643689

[B36] Chen HT Liang S Liao Q Wang S Schumacher JE HIV voluntary counseling and testing among injection drug users in south China: a study of a non-government organization based program AIDS Behav 2007 11 5 778 788 10.1007/s10461-007-9215-x 17347877

[B37] Pang L Hao Y Mi G Wang C Effectiveness of first eight methadone maintenance treatment clinic in China AIDS 2007 21 S8 S103 S107 10.1097/01.aids.0000304704.71917.64 18172377

[B38] Zhao M Wang QY Lu GH Xu P Xu H McCoy CB Risk behaviors and HIV/AIDS prevention education among IDUs in drug treatment in Shanghai J Urban Health 2005 82 S4 iv84 91 16107443 10.1093/jurban/jti110

[B39] Des Jarlais DC Kling R Hammett T Ngu D Liu W Chen Y Binh KT Friedmann P Reducing HIV infection among new injecting drug users in the China-Vietnam cross border project AIDS 2007 21 S8 S109 S114 10.1097/01.aids.0000304705.79541.34 18172378

[B40] Wu Z Luo W Sullian SG Rou K Lin P Liu W Ming Z Evaluation of a needle social marketing strategy to control HIV among injecting drug users in China AIDS 2007 21 S 8 S115 S112 10.1097/01.aids.0000304706.79541.ef 18172379

[B41] Liu B Sullivan GS Wu Z An evaluation of needle exchange programs in China AIDS 2007 21 S 8 S123 S128 10.1097/01.aids.0000304707.56670.cf 18172380

[B42] Lau JT Zhang L Zhang Y Wang N Lau M Changes in the prevalence of HIV-related behaviors and perceptions among 1832 injecting drug users in Sichuan, China Sex Transm Dis 2008 35 4 325 335 10.1097/OLQ.0b013e3181614364 18277942

[B43] Rogers Js Ruefli T Does harm reduction programming make a difference in the lives of highly marginalized, at-risk drug users? Harm Reduct J 2004 1 1 7 10.1186/1477-7517-1-7 15171790 PMC420490

[B44] Watters JK Estilo MJ Clark GL Lorvick J Syringe and needle exchange as HIV/AIDS prevention for injection drug users JAMA 1994 271 2 115 120 10.1001/jama.271.2.115 8264065

[B45] Bluthenthal RN Kral AH Gee L Erringer EA Edlin BR The effect of syringe exchange use on high-risk injection drug users: a cohort study AIDS 2000 14 5 605 611 10.1097/00002030-200003310-00015 10780722

[B46] Millson P Challacombe L Villeneuve PJ Strike CJ Fischer B Myers T Shore R Hopkins S Reduction in injection-related HIV risk after 6 months in a low-threshold methadone treatment program AIDS Educ Prev 2007 19 2 124 136 10.1521/aeap.2007.19.2.124 17411415

[B47] Gowing LR Farrell M Bornemann R Sullivan LE Ali RL Brief report: Methadone treatment of injecting opioid users for prevention of HIV infection J Gen Intern Med 2006 21 2 193 195 16336624 10.1111/j.1525-1497.2005.00287.x PMC1484643

[B48] Merson MH Dayton JM O'Reilly K Effectiveness of HIV prevention interventions in developing countries AIDS 2000 14 S2 S68 S84 11061644

[B49] Perngmark P Vanichseni S Celentano DD The Thai HIV/AIDS epidemic at 15 years: sustained needle sharing among southern Thai drug injectors Drug Alcohol Depend 2008 92 183 190 10.1016/j.drugalcdep.2007.07.014 17870252

[B50] Tang Y Hao W Improving drug addiction treatment in China Addiction 2007 102 1057 1063 10.1111/j.1360-0443.2007.01849.x 17567394

[B51] Des Jarlais DC Marmor M Paone D HIV incidence among injecting drug users in New York City syringe-exchange programmes Lancet 1996 348 987 991 10.1016/S0140-6736(96)02536-6 8855855

[B52] Peak A Rana S Maharjan S Jolley D Crofts N Declining risk for HIV among injecting drug users in Kathmandu, Nepal: the impact of a harm-reduction programme AIDS 1995 9 1067 1067 1070 8527080 10.1097/00002030-199509000-00013

[B53] Remis SR Bruneau J Hankins AC Enough sterile syringes to prevent HIV transmission among injection drug users in Montreal? J Acquir Immune Defic Syndr Hum Retrovirol 1998 18 S1 S57 S59 9663625 10.1097/00042560-199802001-00011

[B54] Norman J Nick M Walsh NN Mugavin J Stoové AM Kelsall J Austin K Lintzeris N The acceptability and feasibility of peer worker support role in community based HCV treatment for injecting drug users Harm Reduct J 2008 5 1 9 10.1186/1477-7517-5-8 18298862 PMC2291043

[B55] Walsh N Walsh N Gibbie MT Higgs P The development of peer educator-based harm reduction programmes in northern Vietnam Drug Alcohol Rev 2008 27 200 203 10.1080/09595230701829348 18264883

[B56] Zhang BZ Xu FH Zhao TY Gao K Li RZ Lin AH Effect evaluating of peer education on AIDS knowledge among withdrawal members after compulsory detoxification Chinese Journal of Health Education 2008 24 578 580

[B57] Luo J Yang F Li HJ Zhu H Peer education on harm reduction in intravenous users in Kunming Chinese Mental Health Journal 2002 16 112 115

[B58] Liua Y Liangb J Zhaob C Zhou W Looking for a solution for drug addiction in China: Exploring the challenges and opportunities in the way of China's new Drug Control La Int J Drug Policy 2009 in press 10.1016/j.drugpo.2009.10.002 19896818

[B59] Ainsworth M Beyer C Soucat A AIDS and public policy: the lesson and challenges of " success" in Thailand Health Policy 2003 64 13 17 10.1016/S0168-8510(02)00079-9 12644326

[B60] Paiva V Ayres JR Buchalla CM Hearst N Building partnerships to respond to HIV/AIDS: non-governmental organizations and universities AIDS 2002 16 S3 S76 S82 10.1097/00002030-200212003-00011 12685928

[B61] Latkin AC Sherman S Knowlton A HIV Prevention among drug users: outcome of a network-oriented peer outreach intervention Health Psychol 2003 22 332 339 10.1037/0278-6133.22.4.332 12940388

[B62] Robles RR Reyes CJ Colón MH Sahai H Marrero CA Matos DT Effects of combined counseling and case management to reduce HIV risk behaviors among Hispanic drug injectors in Puerto Rico: A randomized controlled study J Subst Abuse Treat 2004 27 2 145 152 10.1016/j.jsat.2004.06.004 15450647

[B63] Lyles MC Kay SL Crepaz N Herbst J Passin FW Kim SA Best-Evidence Interventions: Findings From a Systematic Review of HIV Behavioral Interventions for US Populations at High Risk, 2000-2004 Am J Public Health 2007 97 1 133 143 10.2105/AJPH.2005.076182 17138920 PMC1716236

[B64] Schroeder JR Epstein DH Umbricht A Preston KL Changes in HIV risk behaviors among patients receiving combined pharmacological and behavioral interventions for heroin and cocaine dependence Addict Behav 2006 31 868 879 10.1016/j.addbeh.2005.07.009 16085366

[B65] Magura S Siddiqi Q Shapiro J Outcomes of an AIDS prevention program for methadone patients Int J Addict 1991 26 629 655 1757169 10.3109/10826089109058910

[B66] Watters JK Estilo MJ Clark GL Lorvick J Syringe and needle exchange as HIV/AIDS prevention for injecting drug users JAMA 1994 271 115 120 10.1001/jama.271.2.115 8264065

[B67] Schwartz RH Syringe and needle exchange programs worldwide. Part II South Med J 1993 86 323 327 8451673 10.1097/00007611-199303000-00015

